# Kidney transplantation in a patient with HIV disease

**DOI:** 10.4103/0971-4065.53328

**Published:** 2009-04

**Authors:** S. B. Bansal, M. Singhal, R. Ahlawat, V. Kher

**Affiliations:** Department of Nephrology and Kidney Transplantation, Fortis Hospital, Noida, India

**Keywords:** Human immunodeficiency virus, kidney transplantation, antiretroviral therapy

## Abstract

Human immunodeficiency virus (HIV) disease was considered an absolute contraindication to kidney transplantation until recently. The main reason was the concern regarding the side effects of immunosuppressive drugs in already immunocompromised patients. Kidney transplantation is considered to be the best form of renal replacement therapy in most patients with kidney failure. Nowadays, many world medical centers are successfully doing kidney transplantation in HIV patients with kidney failure. However, HIV disease is still considered a contraindication to kidney transplantation in most Indian centers. Here, we report a case of a patient with HIV infection and ESRD, who underwent successful kidney transplantation in our center.

## Introduction

The advent of highly active antiretroviral therapy (HAART) has led to a considerable improvement in the prognosis of patients with HIV.[1] Chronic liver and kidney disease have emerged as important complications, replacing opportunistic infections as the leading causes of death among HIV-infected patients.[1,2] End stage renal disease (ESRD) is common in the context of HIV infection.[3] HIV-associated nephropathy (HIVAN) is the most common cause of ESRD in HIV-infected patients.[4] In the preHAART era, hemodialysis was associated with reduced survival in HIV-positive patients.[5] In the era of HAART however, although the overall survival and morbidity have considerably improved in dialysis patients, it is still far less than in the case of HIV patients who are not on dialysis.[5,6] This has prompted the consideration of renal transplantation for this group of patients. Kidney transplantation is the best treatment for most patients with end stage kidney disease. However, a life expectancy of at least five years is essential before accepting a patient for transplantation.[7] HIV infection was traditionally considered as an absolute contraindication to solid organ transplantation. However, the earlier concerns about additional immunosuppression and its effect on HIV replication, and the risk of further infections have been allayed with increasing experience from a number of centers offering renal and liver transplants to stable HIV-positive patients.[8–14]

According to new guidelines, any HIV patient with end stage renal disease can undergo a kidney transplant if he has stable HIV disease, *i.e*., CD4 cell count >200 cells/*µ*L, HIV RNA copies <50/mL for > 6 months, a stable HAART regimen, and the absence of any AIDS-defining illness.[5] We report here a successful transplantation in an HIV-positive patient on dialysis.

## Case Report

A 66 year-old male from Nigeria was evaluated at Fortis hospital, Noida, in March 2008 for consideration for kidney transplantation. He was known to have diabetes for the last 12 years, hypertension for 5–6 years, and kidney dysfunction in February 2007, and was found to be HIV-positive. He was kept on conservative treatment for kidney disease and started on HAART (a combination of stauvudine 40 mg, lamivudine 150 mg, and nevirapine 750 mg) in March 2007. No kidney biopsy was done and he progressed to ESRD in October 2007 and was subsequently started on hemodialysis. He came to India for a kidney transplant in January 2008 and was investigated and rejected for a kidney transplant in a private hospital. In our hospital, his investigations revealed no HIV RNA and a CD4 cell count of >200 cells. His liver function tests revealed slightly high liver enzymes (ALT: 124, AST: 95). No HCV RNA and HBV DNA were detected. Ultrasound revealed a fatty liver but the serum ceruloplasmin level was normal. The doses of antiretroviral drugs were reduced because these drugs, especially nucleoside reverse transcriptase inhibitors (*e.g.* stauvudine and lamivudine), are known to cause fatty liver and raise aminotransferase levels. Levels of liver enzymes remeasured after two weeks were better (ALT: 74, AST: 54), suggesting that the high liver enzyme levels were due to the antiretroviral drugs. Coronary angiography did not reveal any significant coronary artery disease. Further investigations did not reveal any evidence of opportunistic infections, neoplasm, or any other AIDS-defining illness. The patient underwent a live donor kidney transplantation on 3^rd^ June, 2008; his donor was his son-in-law. Triple immunosuppression therapy comprising of Tacrolimus, Azathioprine, and Prednisolone was initiated one day prior to transplantation; no antibody induction was given. After the transplant, on day 2, he vomited coffee-colored vomitus and had malena. Pantoprazole infusion and Syp. sucralfate were started. Upper GI endoscopy revealed mild gasric erosions but the gastrointestinal bleed subsided with the treatment. On postoperative day 4, the patient developed fever and respiratory distress. He was started on intravenous antibiotics—meropenam and teicoplanin. Blood culture yielded *Klebsiella pneumoniae* that was sensitive to meropenam; it was continued for three weeks. He gradually improved but as his tacrolimus trough level was 14.6 ng/mL on day 4, the tacrolimus dose was reduced. The patient's DJ stent was removed on day 11 and he was discharged on day 14 with a serum creatinine value of 1.3 mg/dL. He subsequently developed leucopenia (TLC = 3.5), therefore, the Azathioprine dose was reduced and later discontinued. CMV PCR and HIV RNA gave negative results and CD4 cell counts were >200 cells/*µ*L. He was continued on double immunosuppression therapy including tacrolimus and prednisolone. Six months after transplantation, he is doing well with a serum creatinine level of 1.0 mg/dL and is on HAART therapy (Tab Triommune 1 twice a day: A combination of stauvudine, lamivudine, and nevirapine). He is presently on double drug immunosuppression treatment including tacrolimus 1.5 mg twice a day and prednisolone 7.5 mg once a day.

## Discussion

This case report illustrates that kidney transplantation may be a good treatment option for stable HIV patients. Our patient had stable HIV disease and was on HAART regimen for > 6 months before transplantation, as revealed by his HIV RNA and CD4 cell counts on 2 occasions 3 months apart, once in February and again in May. He also did not have any opportunistic infection nor had any evidence of neoplastic disease. In absence of kidney biopsy, the most probable cause of CKD in him was HIVAN as evident by rapid progression of kidney disease to end stage renal disease within 6 months time. The only abnormality was raised liver enzymes, but workup did not reveal co infection with any other hepatotropic virus, as is common in these patients and it was most probably a side effect of antiretroviral drugs. His post transplantation course was complicated by chest infection and sepsis, which responded well to antibiotics. He was initially started on triple drug immunosuppression comprising of Tacrolimus, Azathioprine and prednisolone, however one can also use other immunosuppressive drugs like cyclosporine, sirolimus and mycophenolate mofetil. It has been seen that these immunosuppressive drugs have antiretroviral properties.[15] Our patient developed leucopoenia, and azathioprine was stopped, he is doing well on double immunosuppression only. The doses of calcineurin inhibitors (CNIs) have to be decreased because protease inhibitors (PIs) included in the HAART regimen have a strong inhibitory effect on CYP3A4.[15] In this study, the doses of calcineurin inhibitors had been decreased up to 80% when they were used with protease inhibitors. On the other hand, nonnucleotide reverse transcriptase inhibitors (NNRTIs) are CYP3A4 inducers and require higher doses of cyclosporine and tacrolimus. When PIs and NNRTIs are used in combination, the doses of CNIs are reduced to about 50%. Our patient also received the lower doses of tacrolimus and we maintained trough levels of 8–10 ng/mL. We did not give any antibody induction; however, some centers have used induction with IL-2R Ab antagonists in such patients.[9,11]

In the initial report of transplantation in HIV-positive patients by Stock *et al*.,[10] HIV-positive patients underwent kidney transplant and received triple drug immunosuppression therapy consisting of predinisolone, cyclosporine, and Mycophenolate mofetil (MMF) with a mean follow-up of 480 days. The patient survival was 100%, but 5/10 (50%) patients experienced rejections. This was attributed to the dysregulated immune system in HIV-positive patients. The HIV disease remained stable in these patients.[8]

In another report from Drexel University, Philadelphia, 40 HIV patients received kidney transplantation using induction with basiliximab and triple drug immunosuppression consisting of cyclosporine, sirolimus, and steroids. The acturial patient survival rates for years 1 and 2 were 85 and 82% respectively, and graft survival was 75 and 72% respectively, comparable to other high-risk population. The rejection rate was 22%; the HIV disease remained stable.[9]

In the United Network for organ sharing (UNOS) kidney transplant data between 1997 and 2004, graft and patient survival of 38 HIV patients who had received kidney transplants were compared with the survival of 38 HIV-negative recipients who had received a graft from the same donor. The graft survival was higher among HIV- positive patients after five years (76 vs 65%, *P* = 0.21), and patient survival was also higher (91 vs 87% after five years, *P* = 0.72). More grafts failed in HIV-positive patients due to death with functioning grafts rather than rejection. The HIV patients were younger and less often sensitized.[11]

In a recent study by Roland *et al*. who reported one-and three-year outcomes in HIV-infected liver and kidney transplant recipients have shown that one-and three- year patient survival rates were 94% and graft survival was 81% in kidney transplant recipients, similar to HIV-negative patients.[13]

Gruber *et al*. followed eight HIV-positive patients for a median of 15 months (8–47 months) who received induction with basiliximab and triple drug immunosuppression consisting of cyclosporine, mmF, and prednisolone, and achieved a low rejection rate of 13% with excellent patient and graft survival rates (100 and 88% respectively). Cyclosporin dose requirement was very low according to trough level reports.[14]

To summarize, HIV-positive CKD patients with stable disease should not be denied the benefit of kidney transplantation as patient and graft survival is reasonably good, and when monitored closely, the chances of progression of the HIV disease are minimal. The graft and patient survival rates are almost similar in a few studies between HIV-positive and HIV-negative patients.[11,13] Rejections do occur and should be treated with steroids; however, some have also used thymoglobulin to treat vascular rejections.[8] There are still a number of issues that need to be addressed such as long-term patient and graft survival, long-term effects of immunosuppression on CD4 cells, and the pharmacokinetic interactions between antiretroviral drugs and immunosuppressants.
